# Assessment of Wheelchair Propulsion Performance in an Immersive Virtual Reality Simulator

**DOI:** 10.3390/ijerph18158016

**Published:** 2021-07-29

**Authors:** Yu-Sheng Yang, Alicia M. Koontz, Yu-Hsuan Hsiao, Cheng-Tang Pan, Jyh-Jong Chang

**Affiliations:** 1 Department of Occupational Therapy, College of Health Science, Kaohsiung Medical University, Kaohsiung 80708, Taiwan; yusheng@kmu.edu.tw; 2 The Human Engineering Research Laboratories, Veterans Affairs Pittsburgh Healthcare System, Pittsburgh, PA 15240, USA; akoontz@pitt.edu; 3 Department of Rehabilitation Science and Technology, University of Pittsburgh, Pittsburgh, PA 15260, USA; 4 Department of Physical Medicine and Rehabilitation, Kaohsiung Medical University Hospital, Kaohsiung 80756, Taiwan; wannahappy39@gmail.com; 5 Department of Mechanical and Electro-Mechanical Engineering, National Sun Yat-sen University, Kaohsiung 80424, Taiwan; pan@mem.nsysu.edu.tw; 6 Institute of Medical Science and Technology, National Sun Yat-sen University, Kaohsiung 80424, Taiwan

**Keywords:** virtual reality, wheelchair propulsion, spinal cord injuries

## Abstract

Maneuvering a wheelchair is an important necessity for the everyday life and social activities of people with a range of physical disabilities. However, in real life, wheelchair users face several common challenges: articulate steering, spatial relationships, and negotiating obstacles. Therefore, our research group has developed a head-mounted display (HMD)-based intuitive virtual reality (VR) stimulator for wheelchair propulsion. The aim of this study was to investigate the feasibility and efficacy of this VR stimulator for wheelchair propulsion performance. Twenty manual wheelchair users (16 men and 4 women) with spinal cord injuries ranging from T8 to L2 participated in this study. The differences in wheelchair propulsion kinematics between immersive and non-immersive VR environments were assessed using a 3D motion analysis system. Subjective data of the HMD-based intuitive VR stimulator were collected with a Presence Questionnaire and individual semi-structured interview at the end of the trial. Results indicated that propulsion performance was very similar in terms of start angle (*p* = 0.34), end angle (*p* = 0.46), stroke angle (*p* = 0.76), and shoulder movement (*p* = 0.66) between immersive and non-immersive VR environments. In the VR episode featuring an uphill journey, an increase in propulsion speed (*p* < 0.01) and cadence (*p* < 0.01) were found, as well as a greater trunk forward inclination (*p* = 0.01). Qualitative interviews showed that this VR simulator made an attractive, novel impression and therefore demonstrated the potential as a tool for stimulating training motivation. This HMD-based intuitive VR stimulator can be an effective resource to enhance wheelchair maneuverability experiences.

## 1. Introduction

For many individuals with physical disabilities, a manual wheelchair provides the means for independent mobility, better self-care, increased enjoyment of social activities, and empowerment. The use of wheelchairs is not always easy for newly disabled people: they need time to understand and learn how to be confident in using wheelchairs properly and safely for their day-to-day activities. Identifying essential components for training the proper wheelchair propulsion mechanics and wheelchair skills in new manual wheelchair users is an important step in preventing future health and participation restrictions [1]. Maneuvering a wheelchair is an important necessity for the daily life and social activities of people with a range of physical disabilities. Difficulties associated with the use of a wheelchair may reduce independence of users and lead to greater isolation. Previous studies have shown that people with spinal cord injury who are independent in mobility have greater long-term results, like well-being and involvement, than those who are not [2,3,4,5].

Wheelchair training may provide strategies to improve independent wheeled mobility. Further, there is a growing body of evidence supporting the use of accredited manual wheelchair skills training to improve the wheelchair skills capacity of manual wheelchair users [6,7,8,9] and their caregivers [10]. Positive associations have also been established between wheelchair skills and independent mobility [11], functioning [12], and social involvement in manual wheelchair users [4]. Moreover, a higher-level of wheelchair skills could minimize the number of tips and falls in the manual wheelchair, which is the leading cause of injury and death associated with manual wheelchair users [13]. Validated manual wheelchair skills training programs have been successful in developing the wheelchair skills of students and clinicians [14,15,16].

Maneuvering a wheelchair requires complex skills, including vision, balance, strength, spatial perception, and orientation. Like riding a bike, learning how to use a wheelchair properly and safely requires learning. However, due to the short length of recovery, a limited number of basic wheelchair skills were taught upon completion of rehabilitation [17]. In addition, wheelchair navigation can become tiring and exhausting during indoor activities, when the user needs to avoid several obstacles, and during outdoor travel due to inaccessible pathways. In real life, wheelchair users face a variety of common challenges, such as articulate steering, spatial relationships, and negotiating obstacles [18,19], which could all be met in the course of negotiating a single intersection. A novel approach to teaching the wheelchair skills necessary for everyday living is therefore urgently needed.

Virtual reality (VR) is a technology that allows a user to interact with a computer-simulated virtual environment in which multisensory stimuli can be transmitted in a controlled manner. Over the last decade, VR-based systems have been developed to address cognitive, motor, and behavioral disabilities in the fields of assessment, rehabilitation, and training [20,21,22,23]. More precisely, in the context of wheelchairs, VR makes it possible to check the accessibility of public buildings while using a real wheelchair as a means of interaction with the virtual world [24,25] or power wheelchair [26,27,28]. However, much of the previous work using VR simulation used static displays with desktop monitors [29,30] or Cave Automatic Virtual Environment systems [31,32]. These methods did well at providing the experimental control and protection that VR affords but often required high computational requirements and were less realistic than head-mounted display (HMD)-based immersive simulations [33,34]. Unless these conventional systems have a 360-degree field of view, they lack the immersion that the HMD system could provide [35]. Moreover, these displays are expensive and need substantial physical space to setup. On the other hand, the advantage of HMDs is that they combine visual updates with head motion, similar to in real-world experiences, thus providing a more realistic interface for use in training or research. Modern HMDs (e.g., Vive, Oculus) are compact and increasingly affordable as the VR industry moves into the commercial gaming/entertainment sector. Our research group has therefore developed an intuitive VR stimulator for wheelchair propulsion based on HMD, consisting of a support frame with two independent rollers. Users may operate their own manual wheelchairs which are protected by four-point tie-down straps on the platform. It was anticipated that this HMD-based intuitive VR stimulator would make it easier for wheelchair users to achieve similar real-world propulsion performance. Thus, the objectives of this study were to investigate the feasibility and efficacy of our HMD-based intuitive VR stimulator, to compare the performance of wheelchair propulsion between real and virtual tasks using the HMD-based intuitive VR stimulator, and to gather feedback from their subjective preferences. These findings are expected to contribute to knowledge relating to the use of immersive VR research to study wheelchair skills training.

## 2. Materials and Methods

### 2.1. Participants

The study was conducted according to the guidelines of the Declaration of Helsinki, and approved by the Institutional Review Board of Kaohsiung Medical University Chung-Ho Memorial Hospital (KMUHIRB-E(I)-20180076). Informed consent was obtained from all subjects involved in the study. Twenty manual wheelchair users (sixteen males and four females) with spinal cord injuries ranging from T8 to L2 participated in this research. Their mean age and years post-injury were 42.1 ± 9.5 and 13.2 ± 8.4 years, respectively.

### 2.2. Experimental Setup

**Figure 1:**
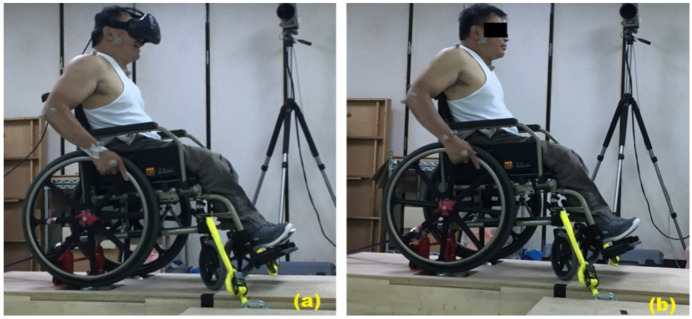
Participant pushed the wheelchair with (**a**) and without HMD (**b**) on a stationary ergometer.

In order to collect their own propulsion performance in nature, participants were asked to push their own wheelchairs, protected by 4-point tie-down straps, on a stationary ergometer at comfort speed for 30 s. The trunk, bilateral upper extremity, and wheel kinematics were obtained using a six-infrared camera motion capture device (Qualisys AB, Gothenburg, Sweden) with 15 reflective markers on the body and the wheel (Figure 1). Subsequently, participants were asked to wear an HMD-based VR (HTC Vive) and underwent 2 min of basic wheelchair maneuvers, such as turning the wheelchair to the left and right, pushing the wheelchair forward a short distance, crossing the intersection of traffic, and driving uphill in a virtual environment. Once they got used to the VR environment, they were asked to operate their own wheelchairs in the following VR scenarios: on level ground at a comfortable speed and in uphill conditions. Three 10 s trials of kinematic data during these two episodes were recorded for the above two conditions and were averaged for further statistical analysis.

### 2.3. HMD-Based Intuitive VR Wheelchair Maneuverability

**Figure 2:**
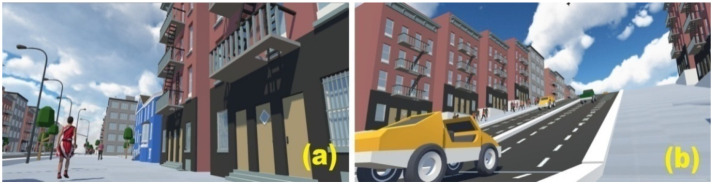
A simulator screenshot of the VR episode at ground level (**a**) and uphill (**b**).

As for the software, database and VR engine, the Unity 3D software was used and coded the virtual wheelchair model, urban street scene, interaction and moving speed between the wheelchair ergometer and the virtual environment, etc. In Figure 2, the images show the graphics that the user can see on the HMD screen. When the participant pushed the handrims to move their wheelchairs to the ground or uphill, the screen showed a person propelling a wheelchair in the same scenario. Participants witnessed a first-person view during the HMD propulsion.

### 2.4. Experimental Setup

A user feedback questionnaire, the Presence Questionnaire (PQ), was used to subjectively test the acceptability and feasibility of this intuitive VR stimulator based on HMD, which consisted of 19 questions in which participants used a 7-point scale to rate various experiences in the VR environment. Participants were also asked to place an “X” on the scale to show their degree of satisfaction. Later on, the maximum overall score is 133 points, suggesting a high level of presence [36,37]. The PQ asked the participants to report their perceptions of the experiences of the virtual environment sensory and control interfaces, their participation in the virtual environment task, the extent and quality of their interactions with the virtual environment, and how easily they adapted to the virtual environment experience. The items assessed various aspects of presence such as involvement/control (sample question: “How much were you able to control events?”), sensory fidelity (sample question: “How closely were you able to examine objects?”), adaptation/immersion (sample question: “How completely were you able to actively survey or search the environment using vision?”), and interface quality (sample question: “How much did the control devices interfere with the performance of assigned tasks or with other activities?”).

Furthermore, the participants also participated in an individual semi-structured interview following the completion of the VR wheelchair maneuvers. The interview questions were extended to the information provided from the participant’s satisfaction and gave them the opportunity to express their views on the VR wheelchair maneuvers in more detail. The interview questions focused on the exploration of VR experience based on participant perspectives (including advantages or disadvantages) and their perception of how the VR wheelchair maneuvers influenced their propulsion pattern. These predefined questions were extended response in PQ and categorized into four aspects: (1) involvement; (2) sensory fidelity; (3) adaptation/immersion; and (4) interface quality. The face and content validity of interview questions were confirmed by a local expert. Interviews were conducted face-to-face by an independent investigator who was not involved in the development of the wheelchair maneuverability simulator or a VR wheelchair maneuvers software, enabling the participants to express their viewpoints in a non-biased manner, and were digitally recorded and then transcribed into documentation. Thematic analysis was used to identify, analyze, and report patterns (themes) that were extracted from the data [38]. Further, critical and representative patterns were extracted from the data and coded into four key themes that indicated how the VR wheelchair maneuverability simulator had immersive experiences in their everyday lives.

### 2.5. Kinematic Data Processing

Data from the wheelchair propulsion experiments were processed with and without a VR stimulator. Each wheelchair propulsion cycle consisted of a push phase and a recovery phase. In present study, the push phase and recovery phase were identified as the hand trajectory (third metacarpophalangeal joint center, MCP3) contacting/leaving the handrims. The start and end push angles were described as the angle between the hand line from MP3 to the rear wheel axle and the horizontal line at rear wheel axle at the start and end of the push phase, respectively. The stroke angle was also described as the difference between the start and the end angles [39,40]. The speed of propulsion was recorded by incremental encoders which were mounted on the rollers and read the rotations of the wheelchair. Shoulder joint angles relative to the trunk were calculated using the local coordinate system approach mentioned in previous studies [41,42]. The relative trunk forward inclination angle (i.e., forward trunk flexion) was determined by measuring the angle between the vertical line drawn from the acromion process through the greater trochanter while sitting at rest in the wheelchair, and the same reference line during the propulsion trials [42]. This is more accurate to reflect the trunk inclination during propulsion in comparison with the sitting posture of the trunk at rest.

### 2.6. Statistical Analyses

Propulsion kinematic variables were averaged for 10 consecutive strokes in the middle of each experiment. A one-way repeated measures analysis of variance, followed by Bonferroni post-hoc comparison test, was used to determine whether the HMD-based intuitive VR stimulator modified the kinematic characteristics of the wheelchair propulsion. Descriptive statistics were used to define the PQ satisfaction level based on the above four aspects of presence: involvement, sensory fidelity, adaptation/immersion, and interface quality. All statistical analyses were performed using the SPSS software (Version 20 for Windows, IBM, Armonk, NY, USA), and the level of significance was set to 0.05.

## 3. Results

### 3.1. The Effect of Immersive VR

The mean start angle of the propulsion (*p* = 0.34), end angle (*p* = 0.46), and stroke angle (*p* = 0.76) were not significantly affected by the immersive VR scenario (Table 1). In either the uphill or ground-level VR episode, spatial similarities of propulsion patterns were found between the scenarios with and without VR. However, when participants propelled their wheelchairs uphill in the VR episode, the speed of propulsion was increased (*p* < 0.01) with a faster cadence (*p* < 0.01). Moreover, they also demonstrated a greater trunk forward inclination (*p* = 0.01) with larger shoulder extension angles (*p* < 0.01), which means that the hand contact moved backward at the start of propulsion. However, no substantial variations in the shoulder range of motion (ROM) were found during propulsion (*p* = 0.66), which indicates that the upper arm movement during propulsion was not significantly influenced by the VR environment.

### 3.2. Subjective Questionnaire and Qualitative Interview

The mean scores of four aspects of presence were 5.48, 5.42, 5.78, and 4.96, respectively (Table 2). During the interviews, participants made similar remarks about the activities, as shown in Table 1. They were also interested in the value of realism in simulator activities for their use in wheelchair skills training. Four themes were created from interview data and were defined as follows.

#### 3.2.1. Theme 1: Involvement Is Important

When participants were asked about their first impression of this VR simulator, all thought it was appealing and attractive. They were able to control VR events, such as propelling forward, or turning in tight spaces, and the responsiveness of the virtual environment to user-initiated action. They indicated that the simulator provided them with immersive interactions in a similar manner.

#### 3.2.2. Theme 2: Sensory Fidelity Is Limited

While this simulator made an impressive and appealing first impression on the participants, a number of critical opinions emerged afterward, particularly when the researcher asked if they thought the VR environment could relate to their previous experiences. Participants felt they were forced to view the street or items presented in the VR environment without any choice and sometimes criticized the quantity and quality of the VR environment as a comedy game.

#### 3.2.3. Theme 3: Benefit Varies in Adaptation/Immersion

When asked whether they thought the simulator was a useful tool in wheelchair skills training, all participants were of the opinion that it could be helpful sometimes, but not always. The simulator performance in helping to learn complex wheelchair skills training might vary depending on the scenario. One concern was that a simulator might be helpful for practicing propelling the wheelchair across the pavement but not for negotiating curbs.

#### 3.2.4. Theme 4: Interface Triggering Does Not Work Well Enough

Most of the participants complained about the quality of the wheeling speed. Participants noticed that it lagged for the first few seconds and then ran smoothly at the start of propulsion. Sometimes the turning direction did not correspond to the driving wheel direction.

## 4. Discussion

This study was a first step toward exploring whether this HMD-based intuitive VR stimulator would make it easier for wheelchair users to achieve similar real-world propulsion performance. In this regard, we found a very similar performance in terms of propulsion speed, cadence, stroke angles, shoulder movement, and trunk posture. In the VR episode with a scenario at surface level, the participants propelled a wheelchair through an interactive, immersive virtual street sidewalk and reported that they quickly adapted to maneuver a wheelchair using a VR stimulator. They were more relaxed pushing their wheelchairs around without worrying about unintentionally bumping into people’s legs and objects.

Moreover, consistent with the findings of previous studies [40,43,44], an increase in propulsion speed and cadence was observed in the VR uphill episode as well as trunk forward inclination. Although our platform was not equipped with an automatic tilt function, the HMD-based intuitive VR stimulator provided an uphill viewing experience. As a result, the participants instinctively bent their bodies and pushed their wheelchairs forward rapidly to keep the uphill momentum going. Previous studies have shown that purely visual signals caused people to adapt their movements to compensate for predicted gravitational shifts (e.g., braking in anticipation of a downhill gravitational boost and exertion in anticipation of uphill gravitational resistance) [45]. However, as no physical slope occurred during VR navigation in our study, participants were unable to perceive the presence of gravitational forces acting on slopes; thus, no significant changes in angles of propulsion and upper arm motion were observed in the present study. This mismatch experience, known as “sensory conflict”, occurred when participants felt their body was moving uphill, but their vestibular system, which is responsible for the spatial orientation and sense of balance, did not receive corresponding information. If a person repeatedly receives sensory information that is different from his or her expectations, the person can experience motion sickness [46]. Some VR users may have troublesome symptoms that are close to motion sickness during VR experiences, which is referred to as VR sickness or cybersickness [47,48]. During our study, some participants reported this discomfort; however, the symptoms disappeared within minutes, leaving no trace. But these unpleasant feelings might inhibit their VR experiences, undermining the average satisfaction score rating of 5 out 7 in subjective questionnaire.

Despite this restriction, the present study showed that this HMD-based intuitive VR stimulator could have the potential to improve the learning motivation of wheelchair users; however, some issues have emerged that need to be considered in order to allow further use. In this study, the simulator made an attractive, novel impression and therefore demonstrated its ability as a tool for stimulating motivation. However, participants pointed out that the training benefits differed according to the scenario. In order to validate this VR simulator, the PQ was used. The average satisfaction rating of 5 out 7 suggested that the participants had more positive impressions of the success of this VR simulator. However, the interface quality received a low performance rating. This finding was consistent with the qualitative results of the interviews. Interface quality items addressed whether control devices or display devices interfere with or distract from task performance and the extent to which participants believed they could focus on the tasks. In recent years, some advanced VR systems have been integrated with motion platforms to provide users with more realistic experiences [49,50]. These motion-coupled VR systems often use servo motors to generate supplementary physical sensations that complement the prevailing visual experiences. These systems provide not only additional features, such as stereoscopy and perspective tracking, but also the missing vestibular sensation. As motion-coupled VR systems with synchronized visual and vestibular cues, these systems could reduce the side effects of cybersickness and provide more comfortable experiences [49]. Despite their many advantages of a virtual environment, these motion-coupled VR systems are too expensive and need significant physical space to setup, making it unlikely to permit this application in a clinical setting. That is why we designed this low-cost HMD-based intuitive VR stimulator. Although this simulator could not fully satisfy all practical needs, it provided functionalities, in particular a learning pathway, which enabled exercises to be proposed that meet the user-specific training needs.

In this study, the HMD-based intuitive VR stimulator used the participants’ wheelchair handrims as the interface in the VR environments instead of the control handles most commonly used with VR software. The encoders were mounted onto the rollers to read the angular velocity and differential wheel velocity between the two wheels for a desired rotation, which facilitated complete immersive handrim propulsion interactions in VR. Therefore, the participants were able to perform similar propulsion patterns in this VR stimulator. However, due to some technical problem, the signal did not provide a fast response for transporting wheel velocities into the virtual environment, which caused low satisfaction for interface quality from the PQ. Future work could be achieved by broadening the hand tracking technology, with a feature that allows the use of hands as an input method to interact in the VR environment. Hence, this will improve the sense of immersion and make the experience even more realistic.

## 5. Conclusions

In conclusion, the ability to practice tasks that are unsafe to practice in the real world is one of the major benefits of VR [23]. Furthermore, VR technology offers a means of practice that is motivational and empowering to the people with disabilities and optimizes goal-directed practice to foster improved learning skills, which can be transferable to many rehabilitation applications (e.g., gait training [51,52], upper limb motor recovery [20], and cognitive rehabilitation [53,54]). It also enhances the clinician’s capacity to evaluate and train locomotion in people with disabilities under conditions that simulate the varied environmental constraints encountered in real life. This study demonstrated the feasibility and efficacy of the HMD-based intuitive VR stimulator for wheelchair propulsion efficiency. This VR simulator offers tremendous benefits, such as safety, motivation, and a variety of experiences of wheelchair maneuverability, and provides a training tool for wheelchair users in a risk-free environment without any physical limitations indoors (e.g., walls, furniture, and stairs) and outdoors (e.g., curb cuts, uneven terrain, and street traffic). Our future work will be to create more VR scenarios and address the hand tracking feature as the input interface in a VR environment. Furthermore, simulations of uneven terrain, including side slope scenarios, and reduction of cybersickness effects are all currently being addressed.

## Figures and Tables

**Table 1:** Propulsion kinematic characteristics for with and without VR conditions.

Propulsion Variable	VR Episode of Uphill	VR Episode on Level Ground	Level Ground without VR	*p*-Value
Speed (m/s)	1.08 ± 0.31 *	0.83 ± 0.20	0.73 ± 0.17	<0.01
Cadence (stroke/s)	1.10 ± 0.29 *	0.88 ± 0.16	0.88 ± 0.24	<0.01
Push time (s)	0.46 ± 0.18 *	0.57 ± 0.14	0.62 ± 0.19	<0.01
Start angle (degree)	101.83 ± 8.64	102.24 ± 12.56	99.30 ± 12.91	0.34
End angle (degree)	29.08 ± 7.69	29.85 ± 8.74	28.29 ± 8.45	0.46
Stroke angle (degree)	72.75 ± 11.14	72.39 ± 15.86	71.01 ± 14.67	0.76
Shoulder extension angle (degree)	55.31 ± 11.12 *	51.54 ± 9.99	51.46 ± 10.04	<0.01
Shoulder ROM (degree)	56.22 ± 7.16	54.82 ± 9.76	55.80 ± 9.82	0.66
Trunk forward inclination angle (degree)	4.66 ± 6.38 *	2.19 ± 3.94	2.67 ± 3.90	0.01

All the values data are expressed as mean ± standard deviation. * indicates significant differences for more than other two conditions by post-hoc pairwise comparisons with Bonferroni adjustments.

**Table 2:** The satisfaction of virtual environment based on the Presence Questionnaire.

*n* = 20	Involvement	Sensory Fidelity	Adaptation/Immersion	Interface Quality
Average satisfaction score (range)	5.48 (4~7)	5.42 (4~7)	5.78 ± 0.61 (5~7)	4.96 (3~6)

## Data Availability

Data available on request due to privacy/ethical restrictions. The data presented in this study are available on request from the corresponding author.

## References

[1] Morgan K., Engsberg J., Gray D. (2017). Important wheelchair skills for new manual wheelchair users: Health care professional and wheelchair user perspectives. Disabil. Rehabil. Assist. Technol..

[2] Fougeyrollas P., Noreau L. (2000). Long-term consequences of spinal cord injury on social participation: The occurrence of handicap situations. Disabil. Rehabil..

[3] Scherer M.J., Cushman L.A. (2001). Measuring subjective quality of life following spinal cord injury: A validation study of the assistive technology device predisposition assessment. Disabil. Rehabil..

[4] Kilkens O.J., Post M.W., Dallmeijer A.J., van Asbeck F.W., van der Woude L.H. (2005). Relationship between manual wheelchair skill performance and participation of persons with spinal cord injuries 1 year after discharge from inpatient rehabilitation. J. Rehabil. Res. Dev..

[5] Krause J., Carter R.E., Brotherton S. (2009). Associations of mode of locomotion and independence in locomotion with long-term outcomes after spinal cord injury. J. Spinal. Cord. Med..

[6] MacPhee A.H., Kirby R.L., Coolen A.L., Smith C., MacLeod D.A., Dupuis D.J. (2004). Wheelchair skills training program: A randomized clinical trial of wheelchair users undergoing initial rehabilitation. Arch. Phys. Med. Rehabil..

[7] Best K.L., Kirby R.L., Smith C., MacLeod D.A. (2005). Wheelchair skills training for community-based manual wheelchair users: A randomized controlled trial. Arch. Phys. Med. Rehabil..

[8] Öztürk A., Ucsular F.D. (2011). Effectiveness of a wheelchair skills training programme for community-living users of manual wheelchairs in turkey: A randomized controlled trial. Clin. Rehabil..

[9] Routhier F., Kirby R.L., Demers L., Depa M., Thompson K. (2012). Efficacy and retention of the french-canadian version of the wheelchair skills training program for manual wheelchair users: A randomized controlled trial. Arch. Phys. Med. Rehabil..

[10] Kirby R.L., Mifflen N.J., Thibault D.L., Smith C., Best K.L., Thompson K.J., MacLeod D.A. (2004). The manual wheelchair-handling skills of caregivers and the effect of training. Arch. Phys. Med. Rehabil..

[11] Mortenson W., Noreau L., Miller W. (2010). The relationship between and predictors of quality of life after spinal cord injury at 3 and 15 months after discharge. Spinal Cord.

[12] Bourbonniere M.C., Fawcett L.M., Miller W.C., Garden J., Mortenson W.B. (2007). Prevalence and predictors of need for seating intervention and mobility for persons in long-term care. Can. J. Aging.

[13] Kirby R.L., Ackroyd-Stolarz S.A., Brown M.G., Kirkland S.A., MacLeod D.A. (1994). Wheelchair-related accidents caused by tips and falls among noninstitutionalized users of manually propelled wheelchairs in nova scotia. Am. J. Phys. Med. Rehabil..

[14] Coolen A.L., Kirby R.L., Landry J., MacPhee A.H., Dupuis D., Smith C., Best K.L., MacKenzie D.E., MacLeod D.A. (2004). Wheelchair skills training program for clinicians: A randomized controlled trial with occupational therapy students. Arch. Phys. Med. Rehabil..

[15] Smith C., Kirby R.L. (2011). Manual wheelchair skills capacity and safety of residents of a long-term-care facility. Arch. Phys. Med. Rehabil..

[16] Best K., Routhier F., Miller W. (2015). A description of manual wheelchair skills training: Current practices in canadian rehabilitation centers. Disabil. Rehabil. Assist. Technol..

[17] Kendall M., Ungerer G., Dorsett P. (2003). Bridging the gap: Transitional rehabilitation services for people with spinal cord injury. Disabil. Rehabil..

[18] Koontz A.M., Bass S.R., Kulich H.R. (2020). Accessibility facilitators and barriers affecting independent wheelchair transfers in the community. Disabil. Rehabil. Assist. Technol..

[19] Meyers A.R., Anderson J.J., Miller D.R., Shipp K., Hoenig H. (2002). Barriers, facilitators, and access for wheelchair users: Sbstantive and methodologic lessons from a pilot study of environmental effects. Soc. Sci. Med..

[20] Henderson A., Korner-Bitensky N., Levin M. (2007). Virtual reality in stroke rehabilitation: A systematic review of its effectiveness for upper limb motor recovery. Top. Stroke. Rehabil..

[21] Laver K., George S., Thomas S., Deutsch J., Crotty M. (2012). Cochrane review: Virtual reality for stroke rehabilitation. Eur. J. Phys. Rehabil. Med..

[22] Piggott L., Wagner S., Ziat M. (2016). Haptic neurorehabilitation and virtual reality for upper limb paralysis: A review. Crit. Rev. Biomed. Eng..

[23] Laver K.E., Lange B., George S., Deutsch J.E., Saposnik G., Crotty M. (2017). Virtual reality for stroke rehabilitation. Cochrane Database Syst. Rev..

[24] Stredney D., Yagel R., Carlson W., Möller T., Shih P.W., Fontana M. Assessing user proficiency through virtual simulations. Proceedings of the RESNA 1997 Conference.

[25] Cooper R.A., Ding D., Simpson R., Fitzgerald S.G., Spaeth D.M., Guo S., Koontz A.M., Cooper R., Kim J., Boninger M.L. (2005). Virtual reality and computer-enhanced training applied to wheeled mobility: An overview of work in pittsburgh. Assist. Technol..

[26] Spaeth D.M., Mahajan H., Karmarkar A., Collins D., Cooper R.A., Boninger M.L. (2008). Development of a wheelchair virtual driving environment: Trials with subjects with traumatic brain injury. Arch. Phys. Med. Rehabil..

[27] Mahajan H.P., Dicianno B.E., Cooper R.A., Ding D. (2013). Assessment of wheelchair driving performance in a virtual reality-based simulator. J. Spinal Cord Med..

[28] Arlati S., Colombo V., Ferrigno G., Sacchetti R., Sacco M. (2020). Virtual reality-based wheelchair simulators: A scoping review. Assist. Technol..

[29] Brooks J.O., Goodenough R.R., Crisler M.C., Klein N.D., Alley R.L., Koon B.L., Logan W.C., Ogle J.H., Tyrrell R.A., Wills R.F. (2010). Simulator sickness during driving simulation studies. Accid. Anal. Prev..

[30] Jack D., Boian R., Merians A., Tremaine M., Burdea G., Adamovich S., Recce M., Poizner H. (2001). Virtual reality-enhanced stroke rehabilitation. IEEE Trans. Neural. Syst. Rehabil. Eng..

[31] Cruz-Neira C., Sandin D., DeFanti T., Kenyon R., Hart J. (1992). The cave: Audio visual experience automatic virtual environment. Commun. ACM.

[32] Repperger D., Gilkey R., Green R., LaFleur T., Haas M. (2003). Effects of haptic feedback and turbulence on landing performance using an immersive cave automatic virtual environment (cave). Percept Mot. Skills.

[33] Shu Y., Huang Y., Chang S., Chen M. (2019). Do virtual reality head-mounted displays make a difference? A comparison of presence and self-efficacy between head-mounted displays and desktop computer-facilitated virtual environments. Virtual Real..

[34] Santos B., Dias P., Pimentel A., Baggerman J., Ferreira C., Silva S., Madeira J. (2009). Head-mounted display versus desktop for 3D navigation in virtual reality: A user study. Multimed. Tools Appl..

[35] Huber T., Wunderling T., Paschold M., Lang H., Kneist W., Hansen C. (2018). Highly immersive virtual reality laparoscopy simulation: Development and future aspects. Int. J. Comput. Assist. Radiol. Surg..

[36] Witmer B.G., Jerome C.J., Singer M.J. (2005). The factor structure of the presence questionnaire. Presence.

[37] Witmer B.G., Singer M.J. (1998). Measuring presence in virtual environments: A presence questionnaire. Presence.

[38] Braun V., Clarke V. (2006). Using thematic analysis in psychology. Qual. Res. Psychol..

[39] Vanlandewijck Y., Theisen D., Daly D. (2001). Wheelchair propulsion biomechanics. Sports Med..

[40] Yang Y.S., Koontz A.M., Yeh S.J., Chang J.J. (2012). Effect of backrest height on wheelchair propulsion biomechanics for level and uphill conditions. Arch. Phys. Med. Rehabil..

[41] Cooper R.A., Boninger M.L., Shimada S.D., Lawrence B.M. (1999). Glenohumeral joint kinematics and kinetics for three coordinate system representations during wheelchair propulsion. Am. J. Phys. Med. Rehabil..

[42] Yang Y.S., Koontz A.M., Triolo R.J., Cooper R.A., Boninger M.L. (2009). Biomechanical analysis of functional electrical stimulation on trunk musculature during wheelchair propulsion. Neurorehabil. Neural. Repair..

[43] Chow J.W., Millikan T.A., Carlton L.G., Chae W.S., Lim Y.T., Morse M.I. (2009). Kinematic and electromyographic analysis of wheelchair propulsion on ramps of different slopes for young men with paraplegia. Arch. Phys. Med. Rehabil..

[44] Gagnon D., Babineau A.C., Champagne A., Desroches G., Aissaoui R. (2015). Trunk and shoulder kinematic and kinetic and electromyographic adaptations to slope increase during motorized treadmill propulsion among manual wheelchair users with a spinal cord injury. BioMed Res. Int..

[45] Porras D.C., Zeilig G., Doniger G.M., Bahat Y., Inzelberg R., Plotnik M. (2020). Seeing gravity: Gait adaptations to visual and physical inclines—A virtual reality study. Front. Neurosci..

[46] Sherman C.R. (2002). Motion sickness: Review of causes and preventive strategies. J. Trav. Med..

[47] McCauley M.E., Sharkey T.J. (1992). Cybersickness: Perception of self-motion in virtual environments. Presence.

[48] Rebenitsch L., Owen C. (2016). Review on cybersickness in applications and visual displays. Virtual Real..

[49] Ng A.K., Chan L.K., Lau H.Y. (2020). A study of cybersickness and sensory conflict theory using a motion-coupled virtual reality system. Displays.

[50] Song S., Yang J. (2017). Configurable component framework supporting motion platform-based VR simulators. J. Mech. Sci. Technol..

[51] De Rooij I., van de Port I., Meijer J. (2016). Effect of virtual reality training on balance and gait ability in patients with stroke: Systematic review and meta-analysis. Phys. Ther..

[52] Porras D., Siemonsma P., Inzelberg R., Zeilig G., Plotnik M. (2018). Advantages of virtual reality in the rehabilitation of balance and gait: Systematic review. Neurology.

[53] Larson E., Feigon M., Gagliardo P., Dvorkin A. (2014). Virtual reality and cognitive rehabilitation: A review of current outcome research. NeuroRehabilitation.

[54] Maggio M., De Luca R., Molonia F., Porcari B., Destro M., Casella C., Salvati R., Bramanti P., Calabro R. (2019). Cognitive rehabilitation in patients with traumatic brain injury: A narrative review on the emerging use of virtual reality. J. Clin. Neurosci..

